# The role of inflammation in hematopoiesis and bone marrow failure: What can we learn from mouse models?

**DOI:** 10.3389/fimmu.2022.951937

**Published:** 2022-08-11

**Authors:** Jun Wang, Miriam Erlacher, Juncal Fernandez-Orth

**Affiliations:** ^1^ Division of Pediatric Hematology and Oncology, Department of Pediatrics and Adolescent Medicine, Faculty of Medicine, University Medical Center Freiburg, University of Freiburg, Freiburg, Germany; ^2^ Faculty of Biology, University of Freiburg, Freiburg, Germany

**Keywords:** hematopoiesis, inflammation, bone marrow failure, mouse models, disease

## Abstract

Hematopoiesis is a remarkable system that plays an important role in not only immune cell function, but also in nutrient transport, hemostasis and wound healing among other functions. Under inflammatory conditions, steady-state hematopoiesis switches to emergency myelopoiesis to give rise to the effector cell types necessary to fight the acute insult. Sustained or aberrant exposure to inflammatory signals has detrimental effects on the hematopoietic system, leading to increased proliferation, DNA damage, different forms of cell death (i.e., apoptosis, pyroptosis and necroptosis) and bone marrow microenvironment modifications. Together, all these changes can cause premature loss of hematopoiesis function. Especially in individuals with inherited bone marrow failure syndromes or immune-mediated aplastic anemia, chronic inflammatory signals may thus aggravate cytopenias and accelerate disease progression. However, the understanding of the inflammation roles in bone marrow failure remains limited. In this review, we summarize the different mechanisms found in mouse models regarding to inflammatory bone marrow failure and discuss implications for future research and clinical practice.

## Introduction

Steady-state hematopoiesis is identified as a hierarchically orchestrated and highly regulated process from embryonic development to adulthood, to produce and replenish the whole blood system from the bone marrow (BM). The hematopoietic homeostasis relies on the division and self-renewing of the hematopoietic stem cells (HSCs) (1), with enormous self-renewal capacities, that differentiate through the lineage-committed progenitors to the different mature blood cells (2). However, severe systematic infections or injuries (e.g. sepsis, chronic inflammatory disease or spinal cord injury) evoking inflammatory signals can give rise to emergency hematopoiesis (3, 4). In this process, pathogens can directly be sensed by pattern recognition receptors, such as Toll-like receptors (TLRs) expressed on hematopoietic stem and progenitor cells (HSPCs) (5). Signaling is then amplified by inflammatory cytokines (interferons (IFN) and tumor necrosis factor (TNF)) secreted at peripheral, immune active sites and/or locally in the BM (6, 7). As a consequence of these stimuli, downstream signaling cascades are initiated and induce the mobilization of HSPCs to replenish consumed short-lived mature hematopoietic immune effector cells and enhance the host defenses (8, 9). Forced repeated cycling of HSCs, however, can result in their exhaustion and eventually in hematopoietic failure, especially when occurring in the context of genetic conditions predisposing to BM failure (BMF). Indeed, elevated levels of IFN and TNF have been found overexpressed in a plethora of syndromes like aplastic anemia (AA) or Fanconi anemia (FA), however, their expression are not sufficient to decipher the pathological role of them between inflammation and disease progression (10–13). Thus, different animal models are helping to identify the link between the specific molecules causing inflammation and BMF, paving the way for novel therapeutic and preventive approaches. In this perspective, we discuss recent findings about how inflammatory signals (including IFN, TLRs, TGF-β, TNF and ILs) as well as inflammatory DNA damage and their interactions can provoke BMF.

## Interferon signaling

Interferons (IFNs) are inflammatory cytokines that were named based on their role in interfering with viral infections (14). They are divided into three different families (I, II and III) with several classes among them. Recently, a new IFN ligand-receptor was identified that may be considered as type IV IFN (15, 16). Type I IFNs are not only released by immune cells upon the interaction with pathogens (i.e. viral, bacterial, fungal, and parasitic infections) but can also be induced endogenously in other cell types by sensing self-ligands, TLR agonists, host factors and cytokines such as TNF (17–25). IFNs are used to clinically treat patients suffering viral diseases and autoimmune diseases as well as different malignancies like chronic myeloid leukemia (CML).

Once IFNs are produced and secreted, they bind to their respective receptors and activate different signal transduction pathways that induce specific transcriptional responses leading to the formation of an antiviral phase in affected cells. For further details, we refer to recent reviews (26–29). Apart from the roles already mentioned, IFNs (principally α and γ) are known to be involved during both developmental and adult hematopoiesis (30, 31). In this sense, IFN-α facilitates embryonic HSC maturation in the aorta-gonad-mesonephros (AGM) during developmental hematopoiesis (32), whereas IFN-γ positively regulates HSC development (33). Beyond their role during developmental hematopoiesis, IFNs are known to play a role as antiproliferative and apoptotic mediators in many different cell types (34, 35). In HSCs, they both induce cell division and impair the self-renewal capacity (8, 9, 36). It has been recently suggested that beside the protective effects of IFNs in the hematopoietic system, they can also compromise the HSPC self-renewal capacity and long-term survival (8, 9, 36, 37), resulting in suppression of blood cell production during adult hematopoiesis (15, 38). Moreover, some studies showed that preservation of murine long-term stem cells (LT-HSCs; defined as Lin^-^,c-Kit^+^,Sca1^+^, CD150^+^,CD48^-^), capable to reconstitute the whole hematopoietic system, is critically compromised by IFNγ (39). This is explained by the fact that IFNγ perturbs thrombopoietin (TPO), inducing phosphorylation of the signal transducer and activator of transcription 5 (STAT5) whose expression is associated with genes implicated in HSC proliferation (37, 39). All these examples show the relevance of the IFN family during infection and immune-mediated processes and the impact of this signaling pathway in HSC differentiation. Due to the important role of IFNs on HSC functions, it is crucial to determine the effects of both chronic and acute IFN exposure on HSCs.

### Chronic interferon signaling results in bone marrow failure

Persistent production of type I and II IFN happening during infection processes has been shown to lead to HSCs exhaustion and eventually, hematopoietic failure (9, 40). This process has been deeply studied through several infection models in mice, which are helping to understand the mechanisms underlying the link between inflammation and BMF to further determine the effect of IFNs on stem cell balance. One of these models is the polyinosinic-polycytidylic acid (poly(I:C)) model, in which a synthetic viral nucleic acid imitates viral infections inducing IFN-α production together with pro-inflammatory cytokines (AP-1 and NF-κB). In 1981, Gidali J. et al.  (41) first tested the effect of type 1 IFN both *in vitro* and *in vivo* on murine HSPCs by analyzing the number of colony forming units (CFU) in S-phase. In both cases, IFN remarkably reduced the number of CFUs without altering the colony subtypes. Although the effect of poly(I:C) is known to mimic the effects of IFN response, it needs to take into consideration that the effects of poly(I:C) cannot be only delimited to IFN itself, as some other signaling pathways are known to be altered. Long-term exposure to type I IFN signaling leads to a functional reduction and finally loss of HSCs in which the IFN-α-dependent transcriptional regulator *Irf2* plays an important role in limiting the excess of IFN signaling  (42). Also in IFN-γ adenylate-uridylate–rich element (ARE)–deleted (del) mice, IFN-γ alone resulted in BMF by disrupting the generation of common myeloid progenitors and lineage differentiation  (43). The poly(I:C) model is not the only model that leads to HSCs exhaustion *via* IFN signaling. Several infections shed light on the influence of IFN on hematopoiesis. *Ehrlichia muris* acute infection changes transiently the activation of LT-HSCs and progenitors from dormancy to activity by IFN-γ signaling in order to induce an innate immune response (44–47). A long-term *Mycobacterium tuberculosis* antigen stimulation leads to a continuous IFN-γ signaling with harmful effects on proliferation and differentiation of HSCs  (48). Similarly, upon recurrent infection with *Mycobacterium avium*, mice become pancytopenic due to a reduction in the number of myeloid-biased HSCs induced by IFN-γ signaling  (36, 40). This myeloid differentiation effect is driven by the overexpression of the *Batf2 *transcription factor* * (36). HSC functional impairment is also observed in the chronic *Lymphocytic choriomeningitis virus* (LCMV) mouse model. Type I and II IFN signaling mediate the depletion of the supportive BM mesenchymal CXCL12-abundant reticular cells network  (49, 50). On the other hand, in both IFN^-/-^ and IFNGR1^-/-^ mice, HCSs show a better reconstitution capability during homeostasis and infectious stress conditions, revealing that IFN-α signaling drives hematopoietic collapse by directly sensitization of HSPCs to undergo cell death and enhanced HSC quiescence (51).

All these models show the relevance of the IFN family during infection processes and the impact of this signaling pathway on HSC differentiation. In addition to the role of IFN in infection models, type I and II IFNs have also a strong impact on BMF syndromes by driving hematopoietic collapse  (52).

Hematopoietic exhaustion caused by chronic IFN-γ signaling is severely accelerated in inherited BMF syndromes (IBMFs). FA is the most common inherited BMF syndrome caused by mutations in one of the 23 genes encoding DNA repair proteins of the Fanconi pathway (53). Several FA mouse models have been established by targeting the disruption of FA genes, such as *Fanca^-/-^*, *Fancc^-/-^*, *Fancg^-/-^* (53–57). In these models, it was demonstrated that FA hematopoietic progenitors are highly sensitive to IFN-γ-induced apoptosis *via* the Fas apoptotic pathway (58, 59). In a model of immune-mediated AA, in which splenocytes were injected intraperitoneally, IFN-γ-dependent HSC loss and hematopoietic failure were driven by macrophages working as sensors of IFN-γ  (60).

Hence, the different animal models developed in the recent years regarding immune-related mechanisms (61–63) and hematopoiesis have helped to understand the pathogenesis of the different congenital and acquired BM failure syndromes (64–67) and might yield further insights into the development of novel therapeutic strategies that will target or even prevent hematopoietic failure in these syndromes.

### Acute interferon signaling can lead to exhaustion of hematopoietic stem cells

In contrast to chronic IFN effects, during acute infections, the production of IFN-γ leads to a transient activation and proliferation of otherwise quiescent HSCs (42, 68, 69), due to the downregulation of different quiescent-enforcing mechanisms (38) among other processes. Indeed, only one single poly I:C injection leading to a short exposure of type 1 IFN, transiently enhances not only HSC proliferation but also early progenitors, followed by quiescence-enforcing mechanisms *in vivo*, helping HSCs to reestablish quiescence, protecting and maintaining so the HSC pool from the IFNs-dependent effects (9, 38).

## TNF signaling

### TNF overexpression is associated with BMF

Another pivotal pro-inflammatory cytokine is the tumor necrosis factor (TNF). It belongs to the TNF superfamily, which consists of 19 different members (70), and is produced by antigen-stimulated macrophages and monocytes. By exerting its function *via* 2 different receptors (TNFR1 and TNFR2), it is responsible for many different signalling processes in the cell, like cellular proliferation, survival, differentiation or apoptosis (71, 72). Among others, it plays important roles in regulating cell functions as immune responses, hematopoiesis and tumorigenesis (73, 74). Although the functions of TNF-α during inflammation are well characterized, the roles during hematopoiesis and HSC homeostasis are poorly described and remain controversial (75–78). From acting as potent inhibitor to promote proliferation, most probably TNF is required for HSC emergence during the development of the embryo after the activation of different signaling pathways, like the activation of the NF-κB pathway *via* TLR4-MyD88 signaling (79). Studies performed in zebrafish revealed that TNF promotes HSC survival and myeloid differentiation by activating a specific p65/NF-κB-dependent gene program that primarily prevents necroptosis (76). Dysregulations on TNF production has been related to directly inhibit growth and induce apoptosis of HSCs, as well as indirectly change the bone marrow microenvironment critical for HSC homeostasis. TNF enhanced expression has been also observed in the pathogenesis of several BMF syndromes, like FA (10, 80–83). Studies using *Fanca^−/−^*, *Fancc^−/−^* and *Fancg^−/−^* mice revealed that TNF production is abnormally high in macrophages, contributing to enhance TNF-induced apoptosis, which relies on the apoptosis signal-regulating kinase 1 (ASK1) (84, 85). Furthermore, HSCs and progenitor cells from *Fancc^−/−^* mice showed that TNF overproduction leads to bone marrow hypoplasia. After long-term exposure, clonal evolution and eventually myeloid leukemia arises secondary to BMF (83). Similarly, by using murine models for immune-mediated AA and genetically modified mice deficient in TNF or TNF receptors it could be observed, that after infusing TNF^-/-^ donor lymph node (LN) cells into CByB6F1 recipients or injection of FVB LN cells into TNFR^-/-^ recipients BM failure was induced. This reveals the importance of this cytokine and these cells in the pathogenesis of the disease (81). Some other studies have revealed that hematopoietic deficiency of the receptor-interacting serine/threonine-protein kinase 1 (RIPK1) results in RIPK3-activation, which leads to necroptosis, and loss of HSPCs and subsequently, to BM failure (86).

## TLR signaling

### Sustained TLRs signals impair HSC function

In the first line of defense of the innate immune system are a family of pattern recognition receptors (PRRs) that detect pathogen associated signatures derived from all kind of microorganisms. In mammals, when PRRs recognize pathogens, they activate downstream a cascade of different signaling pathways by producing IFN1 as well as other mediators in order to display an effective immune response to an acute infection or injury (87). Among the different classes of PRRs, toll-like receptors (TLRs) are known to play a key role in immunity, mediating a rapid inflammatory reaction and appropriate T-cell activation in response to infection and tissue damage. Comprising 10 different members (TLR1-TLR10) in human and 12 in mice (TLR1-TLR10, TLR11-TLR13), TLRs are not only found in most effector immune and stromal cells but also in hematopoietic and progenitor cells as well as endothelial cells (87–91). Besides their well-known job in effector immune cells, TLRs influence HSCs in terms of proliferation and differentiation in response to ‘danger’ signals (e.g. various infections, as well as purified or synthetic TLR ligands induce the release of proinflammatory cytokines like TNF) (92), helping the hematopoietic system to recognize stress events and inducing emergency hematopoiesis (87, 93, 94). Hence, the immuno-surveillance effects of TLRs expressed on HSCs induce the activation of quiescent HSCs pushing them to proliferate and differentiate into myeloid cells. However, a persistent or dysregulated TLR signaling induced by daily injections of LPS for 4-6 weeks affect stem cell balance, leading to ineffective hematopoiesis, loss of HSCs and consequently, to BMF (95). Different mouse models have been developed to further define which bone marrow populations are affected and their relative contribution to the disease pathogenesis (6, 95–98). Some studies revealed TLRs signaling mediated inflammatory pathogenesis in the context of inherited BMF (80). Fanconi gene products protect hematopoietic cells from damage and modulate TLR responses in macrophages (98). TLR8 and the canonical downstream signaling intermediates interleukin 1 receptor-associated kinase (IRAK) and IkappaB kinase-alpha/beta induce TNF production of THP-1 cells and macrophages, which contributes to the hematopoietic defects seen in *Fancc^-/-^* mice (80). Another study on immune-mediated AA suggests that TLR2 and TLR4 individually do not play an essential role in the induction of hematopoietic failure, but depends on IFN-γ and TNF (99). By exposing mice to a TLR-2 agonist, PAM_3_CSK_4_, the augmentation of TLR2 signaling leads to an increase on the phenotype of HSPCs but accompanied with a reduction of bone marrow HSC function (96). Treatment with granulocyte colony-stimulation factors (G-CSF) leads to the induction of TLR expression and signaling resulting in expansion and increase of HSCs but with HSC repopulation defects in mice lacking TLR2, TLR4 or the TLR signaling adaptor MyD88 (98).

## TGF-β and bone marrow failure

In the early 80s, a polypeptide named Sarcoma Growth Factor (SGF) was discovered in transformed rat kidney fibroblasts cultures. This polypeptide was composed by both Transforming Growth Factor-α (TGF-α) and Transforming Growth Factor-β (TGF-β) (100). The TGF-β superfamily comprises a big number of proteins being involved in not only fibroblast growth and collagen production, but also inhibiting cell proliferation among other functions (101, 102). TGF-β has been seen to contribute to hematopoietic suppression in Fanconi Anemia as well as other myelodysplastic diseases (MDS) (103–105). Mice models disrupting TGF-β signaling in FA HSPCs by using a neutralizing antibody called 1D11 (106), significantly improved the proliferation and survival of these cells reducing toxic non-homologous end-joining (NHEJ) machinery and increasing homologous recombinant (HR) activity (107). Indeed, pharmacological inhibition of the TGF-β pathway have shown efficacy in preclinical human and murine models (103, 108, 109).

## Interleukin-1 and -6 and their role in hematopoiesis

Interleukins (ILs) are a group of cytokines with complex immunomodulatory roles produced by leukocytes, lymphocytes and in some particular cases by other type of cells (110). They are involved in many different functions, like proliferation, immune cell differentiation and activation as well playing an important role in the pathophysiology of several disorders (111). ILs comprise more than 50 different members and related proteins, which can be divided into four main groups depending on their structural features. IL-1 is one of the main inflammatory mediators but it is also known to be involved in the regulation of HSCs and HSPCs, including radioprotection, cell growth and/or differentiation as well as altering cell adhesion and migration (112). Indeed, it has been observed that administration of IL-1 prior lethal doses of radiation protects mice from fatal hematopoietic syndrome (113), which is associated to the role of IL-1 on cell cycle activation through the expansion of HSPCs and myeloid precursor cells (114–116). Moreover, chronic IL-1 exposure leads to impairment of HSCs function (117). However, the mechanisms leading to these effects are still largely unknown. Another IL playing a role in hematopoiesis is IL-6. The IL-6 family comprises ten different cytokines: IL-6, LIF, CNTF, CLCF1, OSM, CT-1, IL-11, IL-27, IL-35 and IL-39 (118–120), involved in chronic inflammation, autoimmunity and cancer among other functions (118). Studies have shown that T cells lacking IL-6 led to pancytopenia and BMF as well as deletions on the IL-6 gene were inducing a variable degree of immune-mediated BMF, however, these studies do not indicate a significant or a direct role of IL-6 in murine BMF (99).

### Inflammatory ROS induces DNA damage in HSCs

In response to an increase in the number of inflammatory signals, HSCs are forced to exit their homeostatic quiescent state and proliferate to generate more cells. In this scenario, the chances to acquire and accumulate cellular mutations increase, as more cellular and DNA base pair divisions are happening. Indeed, long-term exposure to IFNα, TLR, or TNF mimicking chronic inflammatory stimuli resulted in an increased mitochondrial reactive oxygen species (ROS)-induced DNA damage in HSCs, which is another mechanism that may cause BMF (52). Moreover, chronic poly(I:C) injections inducing an IFN-I response leads to an increased mitochondrial ROS-induced DNA damage in WT HSCs, resulting in BMF in *Fanca^-/-^* mice with a nonfunctional FA DNA repair pathway (52). In addition, the process of DNA damage in HSCs induced by prolonged LPS stimulation or *Salmonella typhimurium* infection is mediated *via* TLR4-TRIF-ROS-p38 pathway, but not the classic MyD88 signaling (7). TNF-induced accumulation of ROS and oxidative DNA damage leads to premature senescence in HSCs and progenitor cells of WT mice. Furthermore, TNF-treated *Fancc^–/–^* mice showed chromosomal aberrations together with impaired oxidative DNA-damage repair pathway (121). Therefore, it needs to be taken into consideration that all these inflammatory signals can also lead to DNA damage, promoting the depletion of HSCs. In some syndromes defective DNA repair pathways can hence contribute to the pathogenesis of BMF.

## Necroptosis and pyroptosis cause BMF under inflammatory conditions

Programmed cell death (PCD) is an important process that keeps the homeostasis of hematopoiesis among other systems. Being apoptosis and necroptosis the two main forms of PCD, the mode of action of the two processes differs, as apoptosis is not immune-related, while necroptosis leads to inflammation through the secretion of DAMPS (122). As already mentioned, HSPCs respond to DAMPs producing different cytokines to overcome hematopoiesis damage and keep the homeostasis. By the development of several mouse models, it has been observed that an increased in necroptotic death cell in the bone marrow leads to loss of HSPCs while proliferation of SLAM-HSCs happens, inducing stem cell exhaustion and finally BMF (123). Apart from the inflammatory roles of TNF, this cytokine is able to initiate both apoptosis (caspase-8-dependent apoptosis) and RIPK1 kinase dependent necroptosis (124). Several publications have revealed the role of RIPK1 in immune homeostasis and emergency hematopoiesis. Indeed, after an infection induced by an *Ehrlichia* pathogen, RIPK1 is activated, diminishing caspase 8 expression and leading to BMF and hematopoietic suppression after IFNα/β induction (51). Additional work has also shown that IFNα/β is required for the increase of IL-18 expression during infection processes leading to loss of short-term HSCs. Absence of IL-18 was shown to prevent BM aplasia and increase HSCs/HSPCs (125).

Furthermore, it has been shown that RIPK3 plays an important role in generating necroptotic DAMPs as well as promoting the production of inflammatory cytokines (126). Beside apoptosis and necroptosis, Caspase-1 dependent death, also named as pyroptosis, has been shown to trigger HSPCs cytopenias upon NLRP1a inflammasome activation (127). Thus, all these different mechanisms suggest that both necroptosis and pyroptosis might be good therapeutic targets to prevent BMF.

## Clinical therapeutic options - Concluding remarks

**Figure 1:**
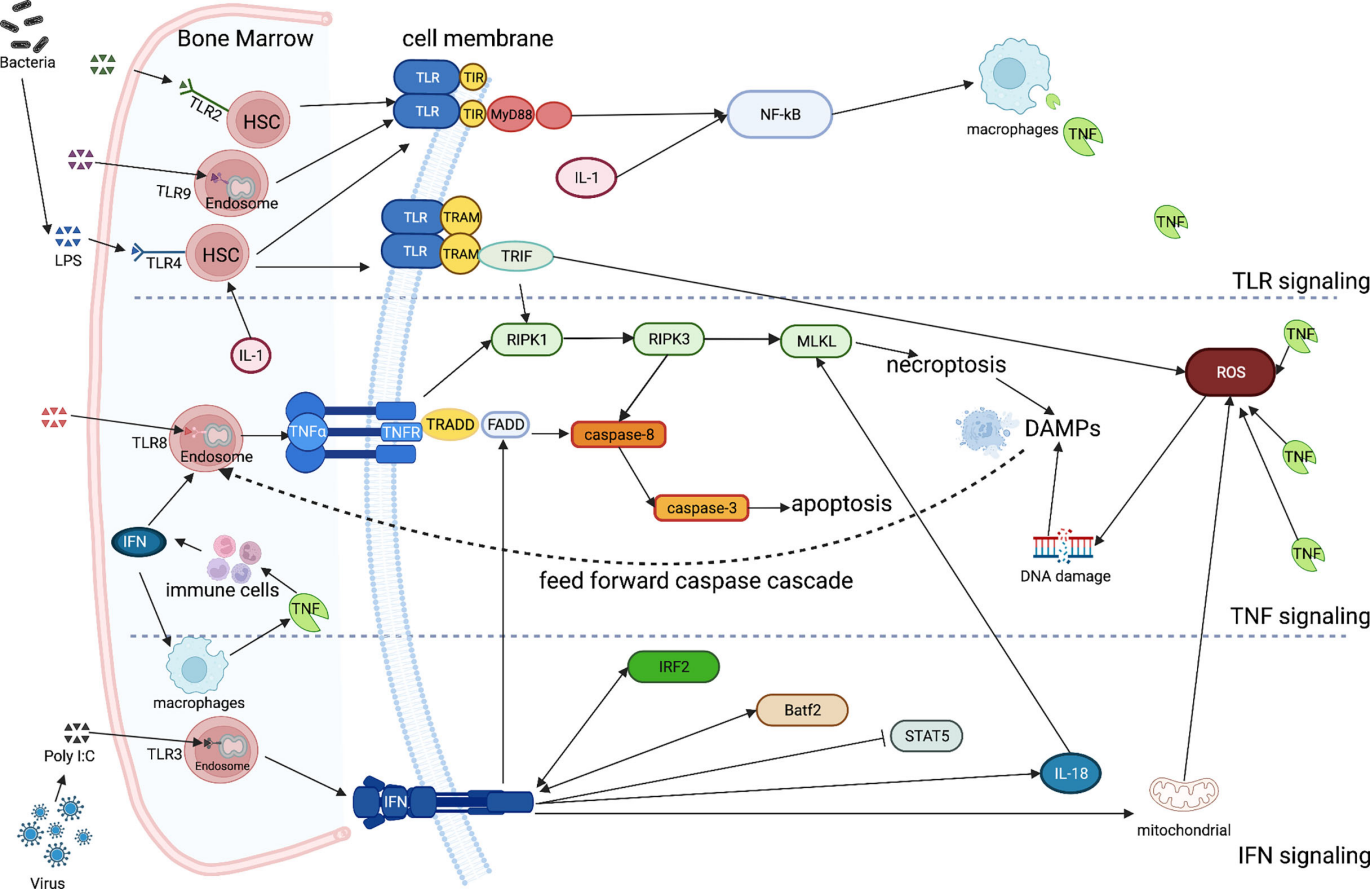
Simplified inflammatory signaling of TLR, TNF, IFN and ILs in bone marrow failure While most of the TLRs are located on the surface of HSCs, some others keep intracellularly (TLR3/8/9). They all directly sense the inflammatory stimuli and induce macrophages to release the excess of TNF through the toll-interleukin-1 receptor (TIR) and the myeloid differentiation primary response 88 (MyD88)-NF-κB pathway. IL-1 directly regulates HSC fate targeting also the NF- κB pathway. TLR4 can specifically activate the TIR-domain-containing the adapter-inducing interferon-β (TRIF) *via* the TRIF-related adaptor molecule (TRAM) cross talking with the TNF signaling, paving the way for necroptosis trough the RIPK1/3- MLKL pathway. The products resulting from necroptosis constitute the DAMPs and feed-forward the caspase cascade. A large amount of TNF may also cause the accumulation of ROS, stimulating immune cells to release IFNs. After the activation of TNFR, TNFR1-associated death domain protein (TRADD) is bound, recruiting the adaptor Fas-associated death domain (FADD) triggering the caspase-8/3 receptor complex, inducing at the end apoptosis. The IFN signal, which is regulated by Irf2 and Batf2, leads also to apoptosis *via* the Fas pathway (FADD), being also perturb the phosphorylation state of STAT5. Moreover, IFN may also lead to an increased mitochondrial ROS, inducing DNA damage. Finally, IFNs are required for an increased in the compromising IL-18 expression, which mediates the MLKL-dependent cell death, compromising hematopoiesis during infection. (Created with BioRender.com).

Altogether, these studies indicate that different inflammatory signaling pathways play an important role in the regulation of hematopoiesis (**Figure 1**, **Table 1**). Long-term exposure to inflammation leads to impairment of HSCs function and self-renew, increasing the chances to develop BMF. Inflammation accelerates hematopoietic failure significantly in mouse models of inherited BMF syndromes such as FA and of immune-mediated AA. It is thus conceivable that inflammatory signals affect the time point of cytopenia onset also in patients with such diseases. It needs to be also mentioned, that all inherited BM failure syndromes as well as immune-mediated AA predispose to leukemia. There is increasing evidence that both infectious diseases and inflammation contribute to the development of hematological neoplasia (128). It is thus conceivable that inflammation does not only promote hematopoietic failure but also consecutive malignant transformation (e.g. by DNA damage induced by ROS). However, a more detailed understanding of the roles of key inflammatory signaling and their interactions in hematopoiesis could open attractive novel ways to develop therapies aimed at modulating the inflammatory immune response to prevent BMF. Antagonizing proinflammatory IFNs, TLR, TNF, and/or ROS may have therapeutic benefits in patients with BMF. The elimination of the key molecules by either neutralizing antibodies or deleting/silencing the genes to abrogated the negative effects of inflammatory factors on HSCs proliferation may lead to restore the ability of the progenitor cells to reconstitute impaired bone marrow, preventing so, fatal consequences derived from BMF. Likewise, inhibition of ROS production may potentially rescue suppressed hematopoietic cell function. For example, Fisetin, a dietary flavonoid, has displayed anti-oxidant activities, which can alleviate CLP-induced multiple organs injury by reducing the expression of TNF and dose-dependently inhibiting the phosphorylation of p38 MAPK, MK2 (129). Rapamycin is an effective therapy in mouse models of immune-mediated BMF by reducing IFN-γ and TNF, stimulating the expansion of functional regulatory T cells, eliminating effector CD8^+^ T cells and preserving hematopoietic stem and progenitor cells (130). Alternatively, downstream effects of inflammation might be targeted. Inhibition of necroptosis and/or pyroptotic cell death might be particularly attractive to prevent hematopoietic failure in inherited BMF syndromes and immune-mediated AA.

**Table 1:** Inflammatory signalling in the mouse models with implication in the bone marrow failure.

Inflammatory signalling/molecules	Disorder/effect(s)	Treatment	Genetically modified mouse	Mechanism(s)	Target cells	Reference(s)
IFN						
ROS	FA, DNA damage	long-term poly I:C injection	*Fanca^-/-^, Sca-1^-/-^, Ifnar^-/-^*	Proliferation and exhaustion of HSCs in CFUs. Increased mitochondrial ROS-induced DNA damage in HSCs	HSC	(41, 42, 52)
		short-term poly I:C injection	*Ifnar^-/-^, WT:Ifnar1^-/-^ BM chimeric*	Enhances HSC proliferation and early progenitors followed by quiescence, helping HSCs to restablish quiescence as well as protecting and maintaining the HSC pool from the IFNs-dependent effects	HSC	(9, 38)
IRF2	HSCs function impairment	long-term poly I:C injection	*Irf2^+/-^, Irf2^-/-^, Irf ^-/-^ Ifnar ^-/-^, Irf2^+/-^ Ifnar ^-/-^*	Impairment of the self-renewal and multilineage differentiation capacity of HSCs	HSC	(42)
IFN-γ	AA		IFN-γ ARE-del	Inhibition of the generation of MPPs and prevention of lineage differentiation	MPP, RBC B cell	(43)
IFN-γ, RIPK1	HSCs function impairment	*Ehrlichia muris* infection	*Ifnγ^-/-^, Ifng^-/-^*	Type I IFN drives HSC/HSPC collapse *via* impaired proliferation and increased RIPK1-dependent cell death during shock-like ehrlichial infection	HSC macrophage	(44–46)
IFN-γ	HSC loss	*Ehrlichia muris* infection	*Ifngr1^-/-^*	IFN-γ effects on macrophages, driving to the loss of HSCs in BM and peripheral HSCs during infection	HSC macrophages	(47)
Batf2	HSCs function impairment	long-term *Mycobacterium tuberculosis* infection		IFN-γ signalling compromises the proliferation and transcriptional program of HSCs	LSK HSC	(48)
Batf2	HSPCs depletion	long-term *Mycobacterium avium* infection	*Ifnar^-/-^*	HSCs and HSPCs are severely depleted displaying IFN-γ signaling-dependent defects in self-renewal	HSC HSPC	(36, 40)
	HSCs function impairment	chronic *Lymphocytic chriomeningitis virus* infection	*Ifnar^-/-^*	Type I and II IFN signaling mediate the depletion of the supportive BM mesenchymal CXCL12-abundant reticular cells network	mesenchymal CXCL12-abundant reticular cells	(50)
	FA		*Fanca^-/-^, Fancc^-/-^, Fancg^-/-^, Fac^-/-^, Fac^+/+^*	FA hematopoietic progenitors are highly sensitive to IFN-γ-induced apoptosis *via* the Fas apoptotic pathway	HSPC MPP	(58, 59)
IFN-γ	SAA	splenocytes intraperitoneal transfer		Macrophages drive HSC loss and hematopoietic failure by working as IFN-γ sensors	HSC Macrophage	(60)
TNF						
ASK1	FA	TNF-α injection	*Fanca^+/-^, Fanca^-/-^, Fancc^-/-^, Fancg^-/-^, Ask1^+/-^, Ask1 ^-/-^*	Macrophages overproduce TNF contributing to the enhancement of TNF-induced apoptosis	HSC HSPC	(83, 84)
	FA	TNF-α injection	*Fanca^-/-^, Fancc^-/-^, Fancg^-/-^, Tnfr1 ^–/–^*	TNF treatment shows chromosomal aberrations together with impairments in the DNA-damage repair pathway	HSC HSPC	(121)
TNFαR	AA, BMF	allogeneic lymph node-cell infusion	*TNF-αR ^-/-^, TNFrsf1a ^-/-^*	TNF from host macrophages and TNFαRs expressed on donor T cells are critical in the pathogenesis of murine immune-mediated	Macrophage T cell BM cells	(81)
RIPK3	BMF		*Ripk1^fl/fl^*, *Ripk3 ^-/-^*	TNF initiates both apoptosis and RIPK1-dependent necroptosis RIPK-dependent necroptosis contributing to BMF	HSC, HSPC LSK, MPP	(86, 123, 124)
TLR						
TLR2 TLR4 ROS TRIF	HSCs function impairment	long term LPS, PAM_3_CSK_4_ injection or *Salmonella typhimurium* infection	*Tlr2 ^-/-^* *Ifnγ ^-/-^* *Tnfα^-/-^*	Ineffective haematopoiesis, loss of HSCs, and consequently, BMF DNA damage in HSCs mediated *via* the TLR4-TRIF-ROS-p38 pathway	HSC HSPC	(7, 95, 96)
	HSCs function impairment	G-CSF injection	*Tlr4 ^-/-^* *Tlr2 ^-/-^* *MyD88 ^-/-^*	Induction of TLR expression resulting in expansion an increase of HSC with HSC repopulation defects in mice lacking TLR2, TLR4 or the TLR signaling adaptor MyD88	HSC	(98)
TLR2 TLR4	AA	allogeneic lymph node-cell infusion	*Tlr2 ^-/-^* *Tlr4 ^-/-^*	TLR2 and TLR4 individually do not play an essential role in the induction of hematopoietic failure, but depend on IFN-γ and TNF	BM cells T cell	(99)
TGF-β						
	HSCs function impairment FA	TGFβ-neutralizing antibody 1D11	*Fancd2 ^-/-^*	Disruption of TGF- β by using a neutralizing antibody improving proliferation and survival reducing NHEJ machinery and increasing the HR activity	HSPC	(106, 107)
	FA	TGFβ-neutralizing antibody 1D11	*Fancd2 ^-/-^*	impaired HSPC function leading to BMF	HSPC	(107)
IL-1						
	HSCs function impairment	chronic IL-1 injection	*Il1r1 ^-/-^*	impairs HSC function not allowing the hyperactivation of PU.1	HSC MPP	(117)
IL-6						
	BMF	pancytopenia	*IL-6 ^-/-^*	T cells lacking IL-6 or a deletion in the gene leads to pancytopenia and BMF and to a variable degree of immune mediated BMF respectively	T cell	(99)
IL-18						
MLKL	HSC/HSPC loss	*Ixodes ovatus Ehrlichia* infection	*Mlk1 ^-/-^*	IL-18 mediated BM aplasia *via* MLKL signaling	HSC HSPC	(125)

## Author contributions

All authors listed have made a substantial, direct, and intellectual contribution to the work and approved it for publication.

## Funding

JW is supported by “Local Excellent Medical Graduated Student” of Shennong Medical Facility. ME received support from the European Research Council (ERC Starting Grant no. 638145 “ApoptoMDS” to ME), the German Federal Ministry of Education and Research (BMBF), Berlin (“MyPred - Network for young individuals with syndromes predisposing to myeloid malignancies” no. 01GM1911A), the EJP-RD program (RiboEurope consortium) and by the Collaborative Research Center (CRC) 1479 “Oncogene-driven immune escape”. JF-O is supported by the Deutsche Forschungsgemeinchaft (no. GZ:FE 2257/1-1).

## Acknowledgments

The authors thank all the members of the group for the critical reading of the manuscript.

## Conflict of interest

The authors declare that the research was conducted in the absence of any commercial or financial relationships that could be construed as a potential conflict of interest.

## Publisher’s note

All claims expressed in this article are solely those of the authors and do not necessarily represent those of their affiliated organizations, or those of the publisher, the editors and the reviewers. Any product that may be evaluated in this article, or claim that may be made by its manufacturer, is not guaranteed or endorsed by the publisher.
